# The perceptual processing of fused multi-spectral imagery

**DOI:** 10.1186/s41235-016-0030-7

**Published:** 2016-12-19

**Authors:** Elizabeth L. Fox, Joseph W. Houpt

**Affiliations:** Department of Psychology, Wright State University, Dayton, 45435 OH USA

**Keywords:** Hyperspectral imagery, Image fusion, Display design, Information processing, Systems factorial technology

## Abstract

Multi-spectral imagery can enhance decision-making by supplying multiple complementary sources of information. However, overloading an observer with information can deter decision-making. Hence, it is critical to assess multi-spectral image displays using human performance. Accuracy and response times (RTs) are fundamental for assessment, although without sophisticated empirical designs, they offer little information about why performance is better or worse. Systems factorial technology (SFT) is a framework for study design and analysis that examines observers’ processing mechanisms, not just overall performance. In the current work, we use SFT to compare a display with two sensor images alongside each another with a display in which there is a single composite image. In our first experiment, the SFT results indicated that both display approaches suffered from limited workload capacity and more so for the composite imagery. In the second experiment, we examined the change in observer performance over the course of multiple days of practice. Participants’ accuracy and RTs improved with training, but their capacity limitations were unaffected. Using SFT, we found that the capacity limitation was not due to an inefficient serial examination of the imagery by the participants. There are two clear implications of these results: Observers are less efficient with multi-spectral images than single images and the side-by-side display of source images is a viable alternative to composite imagery. SFT was necessary for these conclusions because it provided an appropriate mechanism for comparing single-source images to multi-spectral images and because it ruled out serial processing as the source of the capacity limitation.

## Significance

When information from two sensors is for the most part redundant, multi-sensor fusion hinders performance, regardless of whether the images are presented side-by-side or fused into a single composite image. An observer may instead benefit from one single-sensor image that provides the requisite information to make an accurate quick decision. If images from both sensors are displayed, presenting the images side-by-side leads to less inefficient performance than algorithmically fused images. With the particular imagery we used, it is clear that the inefficient performance with the side-by-side imagery is not due to serial processing or to waiting for complete processing of both sources. Instead, the limitation is more likely due to attentional or other intrinsic limitations.

In general, future research on image fusion should include more sophisticated baselines than just performance with single-sensor imagery. Model-based empirical design approaches, particularly systems factorial technology (SFT), illuminate differences in the efficiency with which observers combine information across sensors. Furthermore, SFT can be used to determine whether inefficiencies are due to strategic factors, such as using sensor images in serial checking both images regardless of whether one is sufficient, or due to other intrinsic limitations.

## Background

Information from non-visible parts of the electromagnetic spectrum is beneficial for determining different types of environmental information in many operational settings (Hall & Llinas, 1997). For example, long-wave infrared (LWIR) emissions are useful for detecting heat information (e.g., occluded heat-producing objects, such as a person behind a bush), and short-wave infrared (SWIR; e.g., night vision) can pick up detail in conditions with low illumination. Together, infrared and visible sensors may supply the operator with complementary information and aid in a task such as determining a target’s location (e.g., a person) relative to an object in the scene (Toet, Ljspeert, Waxman, & Aguilar, 1997).

There are several alternative ways to present an observer with multiple sensor images simultaneously. A common family of approaches, which we refer to as *algorithmic* fusion, is to combine relevant information from two sensor images into one composite image (Burt & Kolczynski, 1993). Alternatively, information from each sensor could be displayed in two separate images. Presenting all available information moves the choice of relevant information to the operator rather than relying on an algorithm to detect useful sensor information.

Algorithmic fusion has been the focus of much of the research on presenting multi-spectral information. This is due to two potential benefits of the technique: (1) algorithmic fusion restricts the number of sources of visual information to which the operator must attend and (2) the resultant image may possess emergent features not found in either single image alone (Krebs & Sinai, 2002). A potential downside to algorithmic fusion is that some information from the individual sensors must be filtered out in the process of creating a single image (Hall & Steinberg, 2000). There are many options for algorithmic fusion, and the choice of algorithm does offer some freedom in determining what information is lost, but information is necessarily lost.

In some domains, giving complete information to an operator, particularly expert operators, leads to advantages (cf. Klein, Moon, & Hoffman, 2006). In the image fusion literature, the process of an operator using information from multiple separate images for a task is often referred to as cognitive fusion (cf. Blasch & Plano, 2005) because any potential integration of the two images must take place cognitively. Cognitive fusion is a moniker we will adopt for the rest of this paper. Note that cognitive fusion refers to performance using separate images, not necessarily a particular form of cognitive or perceptual process.

In this paper, we suggest the use of a cognitive-theory-driven approach based on performance, systems factorial technology (SFT), for evaluating image fusion approaches, particularly for comparing algorithmic to cognitive fusion. This approach allows for both more theoretically meaningful measures than raw accuracy or response time (RT), and for insight into the particular aspects of the cognitive process that may have led to better or worse performance. We will begin by briefly reviewing the existing approaches to evaluating image fusion. Next, we review SFT, then apply the methodology to compare algorithmic fusion (in this case Laplacian pyramid fusion, which we describe below) to cognitive fusion (side-by-side image presentation).

### Fusion assessment

Image fusion is mostly studied within the field of computer vision, hence the vast majority of the metrics of fusion quality are based on computational principles. One of the more common measures is of the preservation of edge information, at the individual pixel level (Xydeas & Petrović, 2000); the local, 8×8 pixel grid level (Piella & Heijmans, 2003); or the global image level (Petrović & Xydeas, 2004; Qu, Zhang, & Yan, 2002). These image-level metrics are valuable in that they provide an objective assessment of the amount and quality of information from each single sensor that is represented in the composite image for minimal cost. Two major deficits of limiting assessment to image quality metrics is that they do not account for task-relevant information and are not always predictive of human performance (Smeelen, Schwering, Toet, & Loog, 2014).

To address the shortcomings of computer-based image quality metrics, subjective user experience questionnaires (asking for example, overall reported image preference, comfort, etc.) are used (Krishnamoorthy & Soman, 2010; Petrović, 2007). This approach offers a partial solution, but subjective quality assessments can also fail to predict variation in performance. Furthermore, when they are used, user experience assessments are only used for outcome assessment and not to inform directly the design process (Toet et al., 2010). Hence, while the subjective quality of a display yields some benefits, to gain understanding of what design aspects lead to better decision-making and human performance and inform the design of new fusion approaches, it is important to measure directly human performance on a specific task (cf. Blum, 2006; Dixon et al., 2006; Dong, Zhuang, Huang, & Fu, 2009).

Despite being a relatively limited literature, human performance with fused imagery has been used with a range of basic visual tasks including detection (Krebs et al., 1999), discrimination (e.g., whether a global scene is upright or vertically inverted; Krebs & Sinai, 2002, Toet et al., 1997), recognition (Ryan & Tinkler, 1995; Sinai, McCarley, & Krebs, 1999; Toet & Franken, 2003), and visual search (Neriani, Pinkus, & Dommett, 2008). This research has been conducted in contexts including aviation (Ryan & Tinkler, 1995; Steele & Perconti, 1997) and surveillance (Neriani et al., 2008; Toet & Franken, 2003; Toet et al., 1997). Among these applications, there is a wide range of reported results and overall conclusions. Such discrepancies are potentially due to methodological variation (Ahumada & Krebs, 2000; Essock, Sinai, McCarley, Krebs, & DeFord, 1999; Steele & Perconti, 1997), differences in task descriptions (Krebs & Sinai, 2002; McCarley & Krebs, 2000), and variation in fusion algorithms or sensor combinations (McCarley & Krebs, 2000; Neriani et al., 2008). Additional manipulations often cited in the literature are task type and difficulty, image scene, sensors, and fusion algorithms (Krebs & Sinai, 2002; McCarley & Krebs, 2000). Thus far there is no standard way to compare across manipulations that controls for the amount and type of information provided by each component image.

In many of these studies, performance with composite images was compared to performance with an individual sensor (e.g., LWIR plus visible compared to visible alone). Unfortunately, this comparison confounds whether image fusion enhances performance because of the fusion method implemented or simply because it supplies more information to the observer. We are concerned with answering the question of whether the observer is processing each sensor image as efficiently in a multi-sensor context as when presented in isolation. To answer this question effectively, we must compare performance with multiple sensors to predict how well they should perform given their performance with each individual sensor image.

When an observer is provided with two sensor images, regardless of the display type, they have redundant information to inform them of the correct decision, thereby suggesting an overall faster response. Although it may seem intuitive to equate a performance gain with redundant signals with facilitatory processing, parallel processes with no facilitation can predict significant redundancy gains (Duncan, 1980; Kahneman, 1973; Miller, 1982; Raab, 1962; Townsend & Wenger, 2004). Furthermore, performance decrements may still be observed relative to single-source imagery due to our perceptual system dealing with multiple pieces of information (cf. Townsend & Ashby, 1983; Townsend & Wenger, 2004). Thus, it is important to use an appropriate baseline for assessing the gain (or loss) due to an added signal. The capacity coefficient, a measure from SFT that we describe in detail in the next section, addresses this issue because it uses individual source performance to predict what performance would be in a multi-signal context under a baseline model assumption.

By using SFT, we go beyond the simple better/worse distinctions that are possible with the previously applied metrics. SFT allows us to examine the reason for observed performance differences including the differential effects of increasing the amount of available information (i.e., processing efficiency), facilitation or inhibition between the perception of each source of information, whether processing one image source is sufficient or both sources must be processed, and the temporal organization of the perception (i.e., serial versus parallel).

### Systems factorial technology

To examine the basic perceptual processing of cognitively and algorithmically fused imagery, we applied SFT. The SFT framework supplies information about important cognitive properties including workload capacity, independence, architecture, and the stopping rule. Workload capacity refers to the change in the processing rate of information of an individual sensor when going from single- to multi-sensor presentation. Independence is the degree to which the processing of each type of sensor information influences the processing of the other. Architecture refers to whether processing is simultaneous (parallel processing), sequential (serial processing), or information is pooled (coactive processing). The stopping rule refers to whether one or both sensors must be finished processing when a response is made (e.g., OR or AND).

These SFT constructs are measured using two statistics. The capacity coefficient is used to examine workload capacity and independence. Thus, it is useful for examining how the cognitive processes involved for each source of information (e.g., each sensor image) speed up or slow down as more sources are simultaneously presented (e.g., multiple sensors). The survivor interaction contrast (SIC) is used to examine architecture and the stopping rule, i.e., the SIC is useful for examining the temporal organization of information and the extent to which one or both sensors are processed to completion.

#### Capacity coefficient

The capacity coefficient is the ratio of observed performance with multi-sensor information to a model-based prediction of performance. The model prediction is unique to each individual and task and is based on an individual’s performance with single-sensor images. To predict performance, the model assumes unlimited capacity, and independent and parallel processing (UCIP). The unlimited capacity assumption means that the processing rate of the individual sensor images is the same whether they are presented in isolation or with the other source (cognitively or algorithmically fused). Independent processing indicates that the distribution of processing times for one source does not change based on processing of the other source. Parallel processing indicates that all sensor information is processed simultaneously.

The formal prediction of the UCIP model for OR processing can be stated in terms of the integrated hazard function, *H*(*t*), which indicates the amount of processing completed up to a given time (*t*). For an OR process, the integrated hazard function of the UCIP model is the sum of the integrated hazard functions for each individual process that operates in the parallel system, i.e.,

$$H_{\text{multi-sensor}}^{\text{UCIP}}(t) = H_{\text{visible}}(t) + H_{\text{LWIR}}(t). $$

By using an individual participant’s performance on the visible-only trials to estimate their *H*_visible_(*t*) and likewise for *H*_LWIR_(*t*), we arrive at an individualized estimate of what *H*_multi-sensor_(*t*) would be if that participant were using a UCIP strategy.

The capacity coefficient is the ratio of a participant’s actual hazard function when both sources of information are available to their predicted performance if their processing met the UCIP assumptions:

1 $$ C_{\text{OR}}(t) = \frac{H_{\text{multi-sensor}}(t)}{H_{\text{UCIP}}(t)}.  $$

The numerator of Eq. 1 is the integrated hazard function for multiple sources of information presented simultaneously and the denominator is the summation of the integrated hazard functions of performance for each single source presented in isolation. If *C*(*t*)=1, capacity is classified as unlimited, which occurs if all the UCIP assumptions are met. Deviation from one occurs if one or more assumptions of the UCIP model are violated. *C*(*t*) less than 1, referred to as limited capacity, can occur if processing each source is slower with more sources present (e.g., due to limited attentional capacity), if there is inhibition among the processes, or if processing is serial rather than parallel. *C*(*t*) greater than 1 (super-capacity) implies better performance than a UCIP model and can be due to facilitation between processes including coactive processing.

For inferences regarding the capacity coefficient, we used the standard normal scale statistic (*z*) derived in Houpt & Townsend (2012) to test individual-level deviation from the UCIP model. For a group-level assessment, we applied either *t*-tests or ANOVA to the individual-level *z* scores as appropriate to the hypothesis.

#### Survivor interaction contrast

The SIC is used to examine whether multiple sources of information are processed serially, in parallel, or information is pooled together (coactive) and if one (OR processing) or both (AND processing) sensors are processed to their entirety. Inference based on SICs is done by examining the interaction between slowing down and speeding up cognitive processing of each individual source. We use *S*(*t*) for the survivor function (i.e., the probability that a participant has not responded by a given time) and indicate the level of the salience manipulation by the subscript of *S*(*t*). High salience conditions are denoted H and low salience conditions are denoted L. Throughout this paper, the first subscript indicates the level of the LWIR signal and the second subscript indicates the level of the visible sensor. For example, the survivor function of the RTs when LWIR is high salience and visible is low salience is denoted *S*_HL_(*t*). Using this notation, the SIC is defined as:

2 $$ \text{SIC}(t)= \left[S_{\text{LL}}(t)-S_{\text{LH}}(t)\right]-\left[S_{\text{HL}}(t)-S_{\text{HH}}(t)\right].  $$

**Fig. 1:**
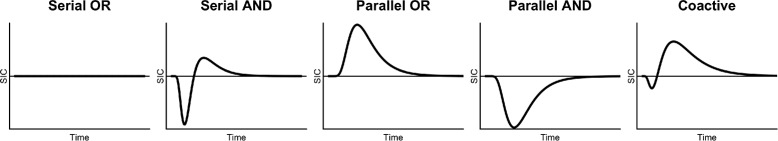
Predicted survivor interaction contrast for parallel, serial, and coactive models with both AND and OR stopping rules

The manipulations that speed up or slow down processing, known as the salience manipulations, must affect only the speed of processing for the respective source of information, a property known as selective influence (Ashby & Townsend, 1980; Dzhafarov, 2003). If the manipulation is effective and selective influence holds, the fastest responses are made when both sources have high salience and slowest when both sources have low salience. If affective selective influence manipulations are used, each of the five classes of models predicts a unique SIC shape (see Fig. 1; Dzhafarov, Schweickert, & Sung, 2004; Houpt & Townsend, 2011; Townsend & Nozawa, 1995; Zhang & Dzhafarov, 2015).

Positive and negative SIC deviations from zero are tested using the Houpt–Townsend statistic (Houpt & Townsend, 2010) and are used to reject candidate processing models. Specifically, the statistic tests for significant deviations from zero of both the largest positive (*D* ^+^) and largest negative (*D* ^−^) value of the SIC curve. If the cognitive process follows a serial-OR rule, the predicted SIC is flat and hence neither *D* ^+^ nor *D* ^−^ should be significant. A parallel-AND model implies an all negative SIC, which should lead to a significant *D* ^−^ but non-significant *D* ^+^. A parallel-OR implies an all positive SIC, hence a significant *D* ^+^ but non-significant *D* ^−^. Both a serial-AND and coactive model result in an SIC that is first negative then positive so both *D* ^+^ and *D* ^−^ should be significant.

Rather than using the traditional conservative cutoff for statistical significance (*α*=0.05), we use *α*=0.33 for our applications of the Houpt–Townsend statistic. Typically, *α* is set to be biased towards indicating a non-significant effect to limit Type I errors. The null hypothesis for the Houpt–Townsend statistic is SIC(*t*)=0 for all *t* and hence conservative *α* levels bias the tests toward indicating a serial-OR signature (flat SIC). While this approach has worked well for model recovery in simulated data (Houpt, 2014), we also applied a recently developed hierarchical Bayesian analysis to the mean interaction contrast (MIC), which we introduce next, to corroborate conclusions from the Houpt–Townsend statistics.

#### Mean interaction contrast

Positive and negative SIC deviations from zero are tested using the Houpt–Townsend statistic (Houpt & Townsend, 2010) and are used to classify the unique processing model; however, these tests can be less statistically powerful than mean level tests because they target distributional level properties. Hence, in some cases it is advantageous to analyze the mean interaction contrast:

3 $$ \text{MIC}(t)= \left[M_{\text{LL}}(t)-M_{\text{LH}}(t)\right]-\left[M_{\text{HL}}(t)-M_{\text{HH}}(t)\right].  $$

MIC predictions for each class of models can be easily derived from the SIC predictions by noting that the integral of the survivor function of a positive random variable is equal to its mean. This implies the area under the curve of the SIC is the MIC. Thus, if processing is parallel (all positive SIC or all negative SIC) then the MIC is nonzero (positive for parallel-OR and negative for parallel-AND). The coactive SIC has both positive and negative ranges, but the positive region is larger, hence the predicted MIC is positive. In contrast, both serial models predict an MIC equal to zero: The serial-OR model has a flat SIC, so the area under the curve is zero. The serial-AND model has both positive and negative regions of the SIC, but they are equal in area so the area under the curve is zero.

The MIC is useful in distinguishing between serial-AND and coactive processes. While both processes imply positive and negative regions of the SIC curve (and, hence, significant *D* ^+^ and *D* ^−^), the coactive model predicts MIC>0, while a serial-AND model implies MIC=0.

A hierarchical Bayesian analysis can estimate a full posterior distribution for both group- and individual-level inferences regarding the MIC (Houpt & Fifić, 2013). Furthermore, this analysis allows for direct comparison between a zero MIC and a positive/negative MIC instead of relying on null-hypothesis significance testing. In this analysis, we used a prior distribution over models in which MIC=0 was the most likely (50%) while MIC>0 and MIC<0 are less likely with equal probability (25%). This prior was based on the assumption that the possible classes of models were equally likely. Serial-OR or serial-AND each imply MIC=0, parallel-OR implies MIC>0, and parallel-AND implies MIC<0. From these analyses, we will report the group-level posterior probability of MIC=0, which we denote $\hat {p}_{\text {posterior}}^{0}$; MIC>0, which we denote $\hat {p}_{\text {posterior}}^{+}$; and MIC<0, indicated by $\hat {p}_{\text {posterior}}^{-}$. We also report the range of individual-level posterior probabilities for each classification of MIC results, positive, negative, or zero.

Although the hierarchical Bayesian approach offers advantages over the Houpt–Townsend statistic, because it focuses on the MIC, it cannot detect the features of the SIC that discriminate between the serial-OR and serial-AND SIC (MIC=0 for both) and between the parallel-OR and coactive predictions (MIC>0 for both). Hence, we report both the Houpt–Townsend statistics and the results of the hierarchical Bayesian MIC analysis below.

### Hypotheses

The use of SFT allows us to examine the underlying processes to help explain why we may see performance benefits of a particular operator display. Each variation in processing structure may inform the cause for a particular pattern of performance. If participants are presented with task-relevant yet redundant information across sensors, they may adopt a processing strategy in which information from only one sensor is used to make the decision (i.e., OR processing or first-terminating). OR processing may combine with either a parallel- or serial-processing structure: either information from both sensors is processed simultaneously but only the fastest to finish is used to make the discrimination (parallel-OR) or information from one sensor is processed and is used for the decision while the alternative sensor is not processed (serial-OR). Alternatively, individual sensor images may each contribute unique complementary information forcing participants to process both sensors entirely to make a correct decision (AND processing). AND processing may also combine with either a parallel- or serial-processing structure: both sensors are processed simultaneously and the slowest to finish is used to make the discrimination (parallel-AND) or both sensors are fully processed, first one, then the other (serial-AND). Fusion also allows for a single percept in which all information is processed in parallel and is pooled to make a decision (coactive processing).

Here we discuss what particular processing mechanisms suggest on a more conceptual level about visual cognition for each presentation type: algorithmic and cognitive fusion.

For algorithmically fused images, standard serial and parallel architectures may be possible, although are a priori unlikely. An interpretation of such a finding would be that participants can selectively attend to one particular spatial frequency information based on the distinctive features to complete the task (Morrison & Schyns, 2001). Alternatively, if observers are unable to extract information selectively from each perceptual dimension, as indicated by McCarley & Krebs (2006), then a coactive or interactive parallel process is more likely (cf. Eidels, Houpt, Pei, Altieri, & Townsend, 2011). For algorithm-fused imagery, we hypothesize: (1) individuals’ efficiency will be at least as high as respective UCIP predictions (i.e., unlimited capacity) across all discrimination stimuli and (2) individuals will use a highly interactive parallel mechanism for processing the multi-sensor information.

When images are presented beside one another (i.e., cognitive fusion), people may process each sensor image in series or in parallel. If processing both images requires visual attention shifts between the two images, then it may be more likely that the images are processed in series. This mechanism limits performance by the constraints of mental integration across several samples of information (Irwin, 1991; Rayner, McConkie, & Zola, 1980). However, serial processes can lead to efficient processing if information from only one image is sufficient for adequate judgments and the additional image is redundant and potentially unnecessary (Neriani et al., 2008).

Alternatively, people may process and potentially integrate the two images in parallel, leaving the opportunity for facilitation in judgment performance due to pictorial redundancy speed-ups (Pollatsek, Rayner, & Collins, 1984), which would imply facilitatory parallel or coactive processing. In contrast, if processing the information across two images is a larger drain on attentional resources, degrading performance with each image (Rousselet, Fabre-Thorpe, & Thorpe, 2002; Scharff, Palmer, & Moore, 2011), inhibitory parallel processing would be observed. Our hypothesis for cognitive fusion focuses on predicting a processing strategy that yields:

1. Performance is no worse than algorithmic fusion. Therefore, individuals’ efficiency will be at least as high as respective UCIP predictions (i.e., unlimited capacity) and across all discrimination stimuli.
2. Individuals will use efficient parallel mechanisms for processing the multi-sensor information.

The cognitive processes involved with utilizing information from multiple sensors may vary from the processing of one sensor image. A cognitively motivated baseline model can encode a specific set of processes so that systematic deviations from the baseline will give evidence for how the processes have changed. Furthermore, using a standardized method to assess deviations of actual performance from predicted performance given the individual parts yields a flexible approach to make comparisons of human processes across several experimental manipulations such as alternative sensors, stimuli, and fusion methods.

## General methods

There was substantial overlap in the methods across the two experiments. In this section, we outline the common methods below then give experiment-specific details in their respective sections.

### Double factorial paradigm

The trials for the SIC were collected in a separate block from those blocks that were included for estimating the capacity coefficient. This allowed us to balance the number of trials in such a way as not to bias responses to one source based on the other source (conditioned on the stimulus) in accordance with the constraints outlined in Houpt et al. (2012) following Mordkoff & Yantis (1991).

To estimate the capacity coefficient, we need RTs from trials in which participants can respond to both visible and LWIR images (i.e., either algorithmically or cognitively fused imagery) as well as trials in which they are focused only on a single source (i.e., visible only or LWIR only). To get the best estimate of what UCIP performance would be, trial type was blocked. Hence, each participant had a block that was entirely dedicated to visible imagery, a separate block dedicated to LWIR imagery, and a block dedicated to fused imagery.

For capacity analyses, we used the imagery without any added noise, which corresponded to the high salience (H) conditions in the SIC analysis (outlined in the “Survivor interaction contrast” section above). Recall, the order of the elements in the subscript indicates the source of information, with the first subscript indicating the LWIR information and the second indicating the visible information. Hence, we denote the visible-only trials with the subscript *∅*H, the LWIR trials with H *∅*, and the fusion trials with HH.

To estimate the SIC, we need RTs from each factorial combination of source image salience (i.e., with or without added noise). To interpret the SIC appropriately, the salience manipulations must satisfy the assumption of selective influence: the presence or absence of noise added to a source image (e.g., LWIR) should affect the perception of that source but not the other source (e.g., visible).

### Participants

All participants self-reported right-handedness, normal or corrected to normal visual acuity, normal color vision, and no difficulties reading English.

### Materials

Stimuli were presented using PsychoPy (Peirce, 2009) on a 20-inch Sony Trinitron monitor. Participants sat at a table 75 cm from the monitor. Responses were made using a right or left click on a two-button mouse.

#### Image collection

The base images were collected using the TRICLOBS three-band night vision system consisting of two digital image intensifiers (Photonis ICUs) and an uncooled LWIR microbolometer (XenICS Gobi 384) constructed by TNO Defense located in Soesterberg, Netherlands (Toet, 2013). The sensor suite registers visual (400–700 nm), near infrared (700–1000 nm), and LWIR (8000–14,000 nm) bands of the electromagnetic spectrum. For this study, we used imagery from the visible and LWIR sensors, as they represent the most distinct ranges of the electromagnetic spectrum in this image set and, hence, potentially carry the most distinctive information.

The optical axes of the three cameras were aligned to minimize the need for registering the images from each sensor post-collection, although further registration was done with software developed by Toet and colleagues (Toet & Hogervorst, 2009). Additional image registration was conducted at the Air Force Research Laboratory. Images were approved for public release (Distribution A: Approved for public release; distribution unlimited. 88ABW Cleared 18 November 2014; 88ABW-2014-5325).

#### Fusion

**Fig. 2:**
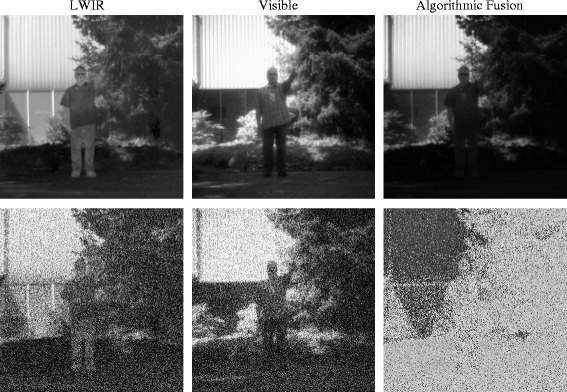
Examples of a LWIR, visible, and algorithmically fused image using the LPT algorithm both with (*bottom images*) and without (*top images*) white noise used for the pointing discrimination stimuli. *LPT* Laplacian pyramid transform, *LWIR* long-wave infrared

We used the Laplacian pyramid transform (LPT; Burt & Adelson, 1983) to combine the visible and LWIR information into one composite image. Subjective and image quality assessments support the use of LPT (Petrović, 2007). LPT is a pixel-level pyramid-based algorithm utilizing six band filters to pass across both sensor images resulting in a series of image components at different resolution qualities. The component images were averaged together across sensors at each band-pass level and combined using a Laplacian transform. The result was a single composite image containing information from both individual sensors (see Fig. 2 for an example).

**Fig. 3:**
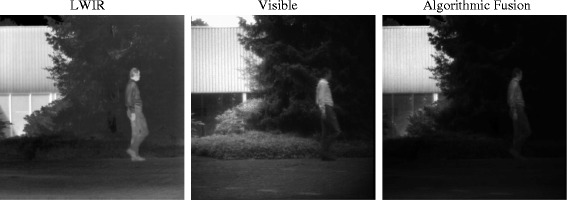
Examples of LWIR, visible, and algorithmically fused images used for the facing discrimination stimuli. In Experiment 1, white noise was added like for Fig. 2. *LWIR* long-wave infrared

Note that, as evident in Figs. 2 and 3, combining a LWIR image and a visible image using the algorithm does not necessarily enhance the image and may actually degrade the quality of the composite representation. Often, added image enhancement techniques are used to provide benefits above raw algorithmic fusion. In our study, we use only the existing algorithm supported in the literature to simulate a more real-world environment where the particular task information, and how to enhance this information further, is unknown before displaying the composite algorithmic image.

#### Salience manipulation for the SIC

To compute the SIC, we needed to selectively speed up and slow down the processing of information for both LWIR and visible images while allowing participants to maintain high accuracy. To reduce the image salience, and hence slow processing, we added zero mean luminance noise to the image. An example of a LWIR image and a visible image with white noise is shown in Fig. 2.

To determine the largest amount of noise that we could add without causing accuracy to drop below 90%, we used the QUEST psychometric method (Watson & Pelli, 1983). Each SIC session began with 120 trials for each single-source image type with varying levels of noise determined by the QUEST adaptive procedure. This allowed us to set individualized salience levels that were specific to each day. Thresholds were estimated each day to account for possible learning and other sources of variation across days. Whether visible only or LWIR only was first was randomly chosen across days and participants.

To compute the SIC, original stimuli (high salience or H) and stimuli with noise (low salience or L) were factorially combined to speed up and slow down the processing of each single sensor. Factorially combining the images led to four unique multi-sensor combinations: high-LWIR + high-visible, high-LWIR + low-visible, low-LWIR + high-visible, and low-LWIR + low-visible. For algorithmically fused trials (Experiment 1 only), the stimulus noise was added before fusing the two images together.

### Procedure

Each experiment consisted of 10 days of 1-hour sessions. All participants were compensated $8 per session with a $2 per session completion bonus: $8 + $2 bonus × 10 days = $100 in total for each experiment.

**Fig. 4:**
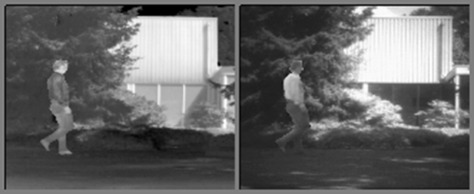
Example of a cognitive fusion presentation of LWIR (always *left*) and visible (always *right*). The participants were asked to discriminate whether the person was facing to their left or right. The two images were centered and presented within 6.39° of visual angle on a mid-gray background. *LWIR* long-wave infrared

The algorithmically fused images were always presented in the center of the screen within 2.86° of visual angle. For cognitive fusion, both single-sensor images were simultaneously presented 0.67° apart (inner-edge to inner-edge) within 6.39° of visual angle on the screen and directly to the left and right of center screen (cf. Fig. 4).

At the beginning of each trial, either a single localization box was shown in the center (algorithmic fusion blocks) or two boxes were presented side by side (cognitive fusion blocks). Localization boxes were always presented for a random interval of time between 400 and 500 msec followed by the stimulus. In the algorithmic fusion blocks, one image was randomly selected and always presented in the middle of the screen. In cognitive fusion blocks, the single-sensor trials required one image that was displayed either to the left or right of the center. In each cognitive fusion trial, the stimuli were displayed with minimal visual angle to allow participants to keep their eyes fixated in the center of the screen without having to saccade for perceptual processing of all the information. Following the stimulus, a blank screen was presented for a response. No trial-by-trial feedback was given.

### Analysis

To analyze differences in operator performance when presented with cognitive or algorithmic fusion, we first applied a traditional analysis of mean correct RTs and accuracy, followed by an SFT analysis. For the SFT analysis, we estimated the capacity coefficient for each individual in each condition. We analyzed the SIC and MIC only for individuals for whom their data did not indicate a violation of selective influence. In the “Results” section, we note whether a participant passed or failed selective influence. To pass selective influence, we used paired Kolmogorov–Smirnov tests of RT survivor distributions to test that, for all *t*: *S*_HH_(*t*)<*S*_HL_(*t*) and $S_{\text {HH}} \ngtr S_{\text {HL}}$, *S*_HH_(*t*)<*S*_LH_(*t*) and $S_{\text {HH}} \ngtr S_{\text {LH}}$, *S*_LL_(*t*)>*S*_HL_(*t*) and $S_{\text {LL}} \nless S_{\text {HL}}$, and *S*_LL_(*t*)<*S*_LH_(*t*) and $S_{\text {HH}} \ngtr S_{\text {LH}}$.

## Experiment 1

In Experiment 1, we investigated the processes underlying the cognitive and algorithmic presentation of two related stimuli, those used for pointing discrimination and those used for facing discrimination. Examples of each sensor image and the combined algorithmic image are shown in Fig. 2 for the pointing discrimination and Fig. 3 for the facing discrimination. We predicted that the facing discrimination stimuli would be more difficult than the pointing discrimination for two reasons: (1) the actor is always located in the center of the image for the pointing condition but in the facing condition the location of the actor varies across trials and (2) the signal in the pointing discrimination stimuli (i.e., entire arm pointing left/right) is more salient than the signal in the facing discrimination stimuli (i.e., contours of the front versus back of the body). This prediction was supported by our findings.

### Method

#### Participants

Ten individuals (six male and four female) participated in this study. Their ages ranged from 20 to 37 years (*M*=25 years).

#### Materials

A total of 2×2×2×10=80 images were used in Experiment 1. There were two types of stimuli (pointing and facing), two sensor images (visible and LWIR) for each scene and an image could either indicate a person pointing (facing) to the left or right. For each direction, there were ten possible scenes (five each of two people). See Fig. 2 for example stimuli. Fusing the visible and LWIR pairs created an additional 40 images.

To reduce image salience, we added zero mean Gaussian luminance noise to the base image before displaying or fusing. Noise samples were independent within and across images.

#### Procedures

Each participant completed 5 days of 1-hour sessions for each stimulus type: pointing and facing (10 days total).

For the first set of stimuli (pointing), participants were asked to discriminate whether a person’s arm was pointing left or right (see Fig. 2). In the second set of stimuli (facing), participants indicated whether a person was facing toward to the left or the right side of the screen (see Fig. 3). If the participant determined left, they pressed the left mouse button, and if right, they pressed the right mouse button. The participants were told to perform the task as quickly and accurately as possible and were informed they must achieve at least 90% accuracy.

The first session of each stimulus type (Day 1: pointing and Day 6: facing) contained trials to compute the capacity coefficient for both cognitive and algorithmic fusion. Based on pilot data, simulations, and time constraints, we collected 120 trials per image type needed for the capacity coefficient (LWIR-alone, visible-alone, and LWIR and visible together). Hence, 360 trials were needed to estimate the capacity coefficient for cognitive fusion and 360 trials were needed to estimate the capacity coefficient for algorithmic fusion for a total of 720 trials.

Other sessions began with 120 trials dedicated to determining the noise level that would lead to 90% accuracy for each image type. This noise level was then used for the low salience images in combination with the original images for the trials required to estimate the SIC. Based on pilot data, simulations, and time constraints, we collected 270 trials per salience condition for a total of 1080 trials per session.

The sessions alternated between algorithmic and cognitive fusion (e.g., Day 2: cognitive fusion, Day 3: algorithmic fusion, Day 4: cognitive fusion, Day 5: algorithmic fusion).

Following the localization box, the stimulus was displayed for 250 msec. Whether the visible image was on the right or the left was randomly varied in cognitive fusion trials. Following the stimulus, a blank screen was presented for 1750 msec allowing the participant 2 seconds to respond starting from stimulus onset.

### Results

In summary, responses were faster and more accurate with visible imagery than LWIR imagery for the pointing discrimination stimuli but the reverse is shown with similar facing discrimination stimuli. Participants had limited capacity with both fusion types, more so for algorithmic than cognitive fusion.

#### Accuracy and mean correct RT analysis

Because the number of sensors could not be fully crossed with fusion type (fused imagery, whether cognitive or algorithmic, included more than one sensor by definition) or with sensor type (when two sensor types were present, then both infrared and visible were necessarily displayed), we computed three separate repeated-measures ANOVA to examine, respectively, single- to multi-sensor comparisons, within multi-sensor comparisons, and within single-sensor comparisons for the mean RT and accuracy.

**Fig. 5:**
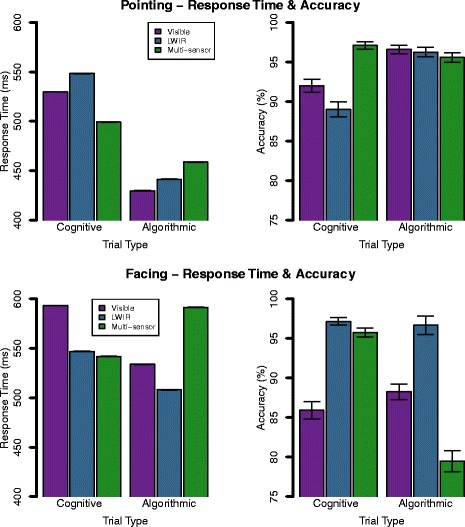
Mean correct RTs (*left*) and accuracy (*right*) for each sensor type for each fusion method in the pointing stimuli (*top*) and the facing stimuli (*bottom*). Cognitive fusion (visible and LWIR alone randomly presented on *left/right* of center) and algorithmic fusion (visible and LWIR alone presented in the *center* of the screen). *Error bars* represent the standard error of the mean (Jarmasz & Hollands, 2009). *LWIR* long-wave infrared, *RT* response time

Table 1 gives the results of a 2×2 repeated-measures ANOVA to assess the effects of the number of sensors presented (single or multiple) and the stimuli (pointing or facing) for both correct RTs and accuracy. For both correct RTs and accuracy, there was a significant interaction between number of sensors and stimuli with the main effects of the number of sensors presented and stimuli type (Table 1). Figure 5 indicates slower, less accurate performance with the facing discrimination stimuli. Across facing and pointing stimuli, performance with multi-sensor imagery suffers more than performance with single-sensor imagery.

**Table 1:** Experiment 1 ANOVA results for the number of sensor images (one or two; visible and LWIR sensors presented alone or simultaneously) and the experimental task (pointing or facing) predicting correct response times and accuracy

	Correct response time			Accuracy		
Condition	*F*	*df*	${\eta _{G}^{2}}$	*F*	*df*	${\eta _{G}^{2}}$
Number of sensors × stimuli	12.45**	1, 9	0.01	60.53***	1, 9	0.19
Number of sensors	5.28*	1, 9	0.00	9.54*	1, 9	0.05
Stimuli	11.57**	1, 9	0.28	17.19**	1, 9	0.36

${\eta _{G}^{2}}$ generalized eta-squared

* *p*<0.05; ** *p*<0.01; *** *p*<0.001

*LWIR* long-wave infrared

Table 2 gives the results of an additional 2×2 repeated-measures ANOVA to assess the effects of the multi-sensor fusion method (algorithmic or cognitive) and the stimuli (pointing or facing) for correct RTs and accuracy. For correct RTs, we found a significant interaction between fusion method and stimuli type with a significant main effect of stimuli. However, we did not find a significant main effect of fusion method (likely due to the cross-over interaction). An analysis of accuracy (Table 2) indicated a significant interaction of fusion type and stimuli with significant main effects of both fusion type and stimuli. Figure 5 indicates algorithmic fusion is faster and slightly less accurate in the pointing discrimination, but is slower and less accurate in the facing discrimination.

**Table 2:** Experiment 1 ANOVA results for the type of fusion technique used to combine the visible and LWIR images (cognitive or algorithmic fusion) and the experimental stimuli (pointing or facing) predicting correct response times and accuracy

	Correct response time			Accuracy		
Condition	*F*	*df*	${\eta _{G}^{2}}$	*F*	*df*	${\eta _{G}^{2}}$
Fusion technique × stimuli	6.73*	1, 9	0.09	56.84***	1, 9	0.43
Fusion technique	0.07	1, 9	0.00	76.17***	1, 9	0.52
Stimuli	13.87**	1, 9	0.27	37.62***	1, 9	0.51

${\eta _{G}^{2}}$ generalized eta-squared

* *p*<0.05; ** *p*<0.01; *** *p*<0.001

*LWIR* long-wave infrared

Lastly, Table 3 gives the results of a 2×2×2 repeated-measures ANOVA to assess the effects of single-image presentation type (left/right of center or center), sensor (visible or LWIR), and stimuli (pointing or facing) to predict correct RTs and accuracy. For both correct RTs and accuracy, the three-way interaction of presentation type, sensor, and stimuli and two-way interaction between presentation type and sensor were not significant. For both correct RTs and accuracy, there was a significant interaction of presentation type and stimuli and a significant interaction of sensor and stimuli with main effects of presentation type and sensor. There was a significant main effect of stimuli for correct RTs but not for accuracy.

**Table 3:** Experiment 1 ANOVA results for the method used to present the single-sensor image to the observer (center of the screen or randomly set to the left or right of center screen) and the type of sensor (visible or LWIR) and the experimental stimuli (pointing or facing) predicting correct response times and accuracy

	Response time			Accuracy		
Condition	*F*	*df*	${\eta _{G}^{2}}$	*F*	*df*	${\eta _{G}^{2}}$
Display method × sensor × stimuli	5.11	1, 9	0.00	2.77	1, 9	0.01
Display method × sensor	2.04	1, 9	0.00	0.11	1, 9	0.00
Display method × stimuli	6.92*	1, 9	0.04	7.29*	1, 9	0.07
Sensor × stimuli	32.58***	1, 9	0.05	32.56***	1, 9	0.28
Display method	92.53***	1, 9	0.26	6.46*	1, 9	0.08
Sensor	6.15*	1, 9	0.00	40.46***	1, 9	0.13
Stimuli	8.93*	1, 9	0.22	2.11	1, 9	.04

${\eta _{G}^{2}}$ generalized eta-squared

* *p*<0.05; ** *p*<0.01; *** *p*<0.001

*LWIR* long-wave infrared

Recall that for algorithmic fusion blocks, the single-sensor image was always presented in the middle of the screen. In cognitive fusion blocks, the single-sensor trials required one image that was displayed either to the left or right of the center. Figure 5 indicates both LWIR and visible single-sensor trials were faster and more accurate when visual attention was anticipating stimuli on a smaller visual area (algorithm-fused block of trials) than a larger visual area (cognitive-fused block of trials) even though the same single-sensor image was presented in both conditions.

#### SFT analysis

Further individual-level analyses of the capacity coefficient and the SIC allows us to examine how cognitive processing changes across the manipulated fusion type, sensor, and stimuli conditions by participant. Separate analyses of SFT were conducted for algorithmic and cognitive fusion across both pointing and facing stimuli for those who satisfy selective influence. We first report these results for the pointing stimuli, then the facing stimuli.

In the pointing stimuli, the capacity coefficient function was below 1 (i.e., limited capacity) for some time for both cognitive and algorithmic fusion for all participants. Individual capacity *z* scores in the pointing stimuli ranged from −9.5 to −6.4 for algorithmic fusion and from −4.2 to 0.08 for cognitive fusion (Table 4). The performance hypotheses were supported at the group level: we found limited workload capacity across both fusion types [algorithmic fusion *t*(9)=−28.36, *p*<.05, *d*=12.68; cognitive fusion *t*(8)=−3.59, *p*<.05, *d*=1.69]. Algorithmic fusion was significantly more limited than cognitive fusion [ *t*(8)=8.54, *p*<.05, *d*=3.99].

**Table 4:** Experiment 1: Individual-level capacity, *z* score, and statistical significance for algorithmic and cognitive fusion of multi-sensor images compared to the UCIP model in the pointing discrimination stimuli

	Algorithmic		Cognitive	
Subject	Capacity	*z* score	Capacity	*z* score
1	Limited	−8.174***	N/A	N/A
2	Limited	−6.367***	Unlimited	−0.088
3	Limited	−8.182***	Unlimited	−0.653
4	Limited	−7.694***	Limited	−4.056***
5	Limited	−7.780***	Unlimited	0.088
6	Limited	−9.155***	Limited	−3.322***
7	Limited	−7.436***	Limited	−4.219***
8	Limited	−7.547***	Limited	−4.066***
9	Limited	−7.660***	Limited	−2.362*
10	Limited	−9.500***	Unlimited	−0.826

*N/A* not applicable, *UCIP* unlimited capacity, and independent and parallel processing

* *p*<0.05; ** *p*<0.01; *** *p*<0.001

For SIC analyses of cognitive fusion, selective influence could not be rejected for six participants based on a series of Kolmogorov–Smirnov tests. The Houpt–Townsend SIC statistic (Houpt & Townsend, 2010) indicated three participants had a significant positive SIC, one participant had a significantly negative SIC, and two participants had neither significant positive nor significant negative deviation, but did have a significantly positive MIC. Recall that a significance cutoff of *α*=.33 was used for the SIC and MIC tests. The remaining four participants failed tests of selective influence precluding the interpretation of their SICs. Table 5 lists each participant’s Houpt–Townsend SIC statistic for both positive and negative deviations from zero, the MIC statistic, and the corresponding processing model.

**Table 5:** Cognitive fusion results of the pointing stimuli in Experiment 1 including whether the participant (for a particular day) passed the test of selective influence, the Houpt–Townsend statistic (*D* ^+^ and *D* ^−^), the mean interaction contrast (MIC), and the identified processing model

Subject	Pass/fail	*D* ^+^	*D* ^−^	MIC	Architecture
4	Pass	0.018	**0.131** ^+^	**-61.912** ^+^	Parallel-AND
5	Pass	**0.180** ^+^	0.065	15.943*	Parallel-OR
6	Pass	**0.179** ^+^	0.055	**9.09** ^+^	Parallel-OR
8	Pass	**0.159** ^+^	0.073	**25.321** ^+^	Parallel-OR
9	Pass	0.096	0.086	**12.667** ^+^	Ambiguous
10	Pass	0.101	0.011	**37.663** ^+^	Ambiguous

*MIC* mean interaction contrast

Houpt–Townsend statistic: ^+^ *p*<0.33; * *p*<0.05; ** *p*<0.01; *** *p*<0.001

MIC: ^+^ *p*<0.33; * *p*<0.05; ** *p*<0.01; *** *p*<0.001

Bold *D* ^+^ and *D* ^−^ statistics indicate a significant Houpt–Townsend statistic at *p*<0.33

Using the hierarchical Bayesian model, we found minimal evidence for a zero MIC at the group level ($\hat {p}_{\text {posterior}}^{0}=.52$). The remaining models were unlikely ($\hat {p}_{\text {posterior}}^{-}=0.19$; $\hat {p}_{\text {posterior}}^{+}=0.29$). At the individual level, the ratio of posterior odds (i.e., most likely model divided by the second most likely model) did not show strong evidence of a particular processing architecture and stopping rule for any individual. The ratio of posterior odds ranged from 1.49 to 2.42. Note that using the Kass & Raftery (1995) scale, a ratio less than 3.2 is considered insufficient evidence from which to draw strong conclusions.

For algorithmically fused images, no participant’s data satisfied the assumptions of selective influence, thereby precluding the use of the SIC for model classification.

With the facing stimuli, Participant 1 did not obtain at least 80% accuracy in all conditions for further analysis of workload capacity with multi-sensor information. For other participants, *C*(*t*)<1 for some time for both cognitive and algorithmic fusion. Capacity *z* scores ranged from −10.7 to −8.5 for algorithmic fusion and from −4.9 to −2.2 for cognitive fusion (Table 6). We hypothesized that individuals’ efficiency for both algorithmic and cognitive fusion was at least as high as respective UCIP predictions (i.e., unlimited capacity) for the facing discrimination stimuli. The performance hypotheses were not supported at the group level; we found limited workload capacity [ *C*(*t*)<1] across both fusion techniques [algorithmic fusion *t*(8)=−45.80, *p*<.05, *d*=21.59; cognitive fusion *t*(9)=−14.32, *p*<.05, *d*=6.40] with algorithmic fusion significantly more limited than cognitive fusion [ *t*(8)=14.30, *p*<.05, *d*=7.24].

**Table 6:** Experiment 1: Individual-level capacity, *z* score, and statistical significance for algorithmic and cognitive fusion of multi-sensor images compared to UCIP model in the facing discrimination stimuli

	Algorithmic		Cognitive	
Subject	Capacity	*z* score	Capacity	*z* score
1	N/A	N/A	Limited	−3.992
2	Limited	−9.586***	Limited	−3.985***
3	Limited	−9.137***	Limited	−3.985***
4	Limited	−8.597***	Limited	−4.757***
5	Limited	−9.702***	Limited	−4.879***
6	Limited	−10.748***	Limited	−3.459***
7	Limited	−9.517***	Limited	−4.515***
8	Limited	−8.980***	Limited	−4.189***
9	Limited	−10.036***	Limited	−4.296***
10	Limited	−9.750***	Limited	−2.676**

*N/A* not applicable, *UCIP* unlimited capacity, and independent and parallel processing

* *p*<0.05; ** *p*<0.01; *** *p*<0.001

We divided individuals’ data into two separate days to compute the SIC because no one participant passed the tests of selective influence when combining across days. For cognitive fusion SIC analyses, selective influence was not rejected for four participants for one of the two days of data collection. All four participants’ SIC function had no significant deviations from zero. Table 7 lists each participant’s Houpt–Townsend SIC statistic for both positive and negative deviations from zero, the MIC statistic, and the processing model that would predict that pattern of significance.

**Table 7:** Cognitive fusion results of the facing stimuli in Experiment 1 including whether the participant (for a particular day) passed the test of selective influence, the Houpt–Townsend statistic (*D* ^+^ and *D* ^−^), the mean interaction contrast (MIC), and the identified processing model

Subject	Pass/Fail	*D* ^+^	*D* ^−^	MIC	Architecture
6.2	Pass	0.154	0.075	16.839	Serial-OR
7.2	Pass	0.136	0.123	40.692	Serial-OR
8.1	Pass	0.118	0.110	6.251	Serial-OR
9.2	Pass	**0.192** ^+^	0.069	4.310	Ambiguous

*MIC* mean interaction contrast

Houpt–Townsend statistic: ^+^ *p*<0.033; * *p*<0.05; ** *p*<0.01; *** *p*<0.001

MIC: ^+^ *p*<0.033; * *p*<0.05; ** *p*<0.01; *** *p*<0.001

Bold *D* ^+^ and *D* ^−^ statistics indicate a significant Houpt–Townsend statistic at *p*<0.33

Using the hierarchical Bayesian model, we found minimal evidence for a zero MIC at the group level ($\hat {p}_{\text {posterior}}^{0}=.54$). The remaining models were unlikely ($\hat {p}_{\text {posterior}}^{+}=0.28$; $\hat {p}_{\text {posterior}}^{-}=0.18$). All participants’ most likely model was MIC=0 and the second most likely model MIC>0. For these participants, the ratio of posterior odds ranged from 1.62 to 2.63 indicating very weak evidence for each individual. Thus, for both individual- and group-level conclusions, we found weak evidence for a serial processing model. These results are consistent with SIC findings of no significant deviations from zero.

As with the pointing, for algorithmically fused images, no participant’s data satisfied the assumptions of selective influence, thereby precluding the use of the SIC for model classification.

We used a repeated-measures ANOVA to examine the effects of stimulus (pointing or facing) and fusion type (cognitive or algorithmic) on capacity *z* scores. The interaction was non-significant [ *F*(1,8)=0.03, *p*=0.87, and ${\eta _{G}^{2}} = 0.00$], and the main effect was significant for both stimulus type [ *F*(1,8)=20.53, *p*<.05, ${\eta _{G}^{2}} = 0.37$] and fusion type [ *F*(1,8)=137.94, *p*<.05, and ${\eta _{G}^{2}} = 0.87$]. Capacity *z* scores with the pointing stimuli were higher than *z* scores with the facing stimuli for both fusion types, with algorithmic fusion more limited than cognitive fusion.

### Discussion

Both cognitive and algorithmic fusion hindered processing of the individual source images relative to independent parallel processing. Because information was redundant across the two images, participants should be faster with two images than with a single image, even with independent parallel processing of each image (cf. Raab, 1962). Subjects were slightly faster with the side-by-side images than the single-source images; however, the capacity results indicate that the speed-up was not as much as would be observed from independent parallel processing. Performance was even worse with the algorithmically fused images: RTs were slower with algorithmically fused images than with either of the single-sensor images. Hence, capacity coefficient values were quite low for algorithmic fusion, much lower than cognitive fusion.

Low capacity coefficient values can result from a number of different violations of the baseline UCIP model predictions. All other factors being equal, serial processing systems have more limited capacity than parallel, while coactive processing systems have higher capacity than standard parallel (Townsend & Nozawa, 1995; Townsend & Wenger, 2004).^1^ Unfortunately, our results from the SIC analysis did not lead to clear results regarding processing architecture. All participants’ data indicated violations of selective influence for the algorithmically fused images. Most participants indicated a violation of selective influence with cognitive fusion. Of those participants that did not violate the distribution ordering implied by selective influence, null-hypothesis testing indicated a variety or processing strategies: parallel-OR process and parallel-AND with the pointing stimuli and serial-OR with the facing stimuli. The Bayesian analysis of the MIC indicated that there is very slight evidence in favor of a zero MIC at the group level (MIC=0) and similarly minimal evidence for any MIC category (positive, negative, or zero) at the individual level for both stimuli types.

Among those participants wo may be using a parallel-OR processing strategy, capacity coefficients were still quite limited indicating that there may be other deficits relative to the UCIP model. Given the short presentation time and that at least one of the images was extrafoveal, a violation of the unlimited capacity assumption is a likely cause. With a single image, participants can fixate on the most informative region of that image to get the most out of the image. When there are two images, at most one can be fixated so information uptake is almost certainly not the same with two images relative to one. Limitations of visual short-term memory may degrade the ability to integrate information from multiple sensors or potentially facilitate the strategy to process only a single informative sensor image (Irwin, 1991; Rayner et al., 1980).

With algorithmic fusion, only a single image is presented, so participants can fixate the most informative region. Hence, the limitations on visual attention that may explain low capacity values for cognitive fusion are not sufficient for algorithmic fusion. Although we were not able to draw direct inferences from the SIC, we can make some inferences about the processing. Independent serial or parallel processing are unlikely candidates, as they should have led to effective selective influence and hence ordered distributions (Dzhafarov, 2003; Houpt & Townsend, 2010; Houpt, Blaha, McIntire, Havig, & Townsend, 2014). A priori, it is difficult to imagine how (or why) the visual system would separate the information from each source before processing. Indeed, previous research using sophisticated accuracy-based methodologies found that individual sensor information was perceptually nonseparable in an algorithmically combined image (McCarley & Krebs, 2006). Because the combined algorithmic image is processed as a single unit of information that integrates information from both sensors, the visual processing decision is like a coactive process. However, unlike most coactive processes, the capacity values are much lower than independent parallel, not higher. This suggests that useful information is lost in the fusion process, perhaps more akin to an inhibitory parallel process (cf. Eidels et al., 2011). The potential information loss is evident in Figs. 2 and 3, in which the person looks more clearly differentiated from the background in the single-sensor images than in the algorithmically fused image.

Based on McCarley & Krebs (2000) and Krebs & Sinai (2002), we had assumed that a more difficult stimulus set (i.e., degraded quality of image and type of psychophysical task) would lead to higher capacity coefficient values for the algorithmically fused imagery. The more difficult stimuli in our experiment were when the facing stimuli were not directly centered (since the pointing stimuli were centered) and there were fewer physical cues to aid in decision-making. Capacity was higher at the group level with the pointing stimuli than with the facing stimuli when using algorithmic fusion (as well as cognitive fusion), although it was not enough of an increase to reach the capacity values from cognitive fusion, let alone the predicted UCIP baseline.

There was some evidence of a differential speed–accuracy trade-off between the algorithmically fused imagery and the cognitively fused images. Algorithmic fusion led to faster and slightly less accurate performance than cognitive fusion in the pointing stimuli. However, algorithmic fusion led to both slower and much less accurate performance than cognitive fusion in the facing stimuli. This may suggest that different fusion approaches may be more appropriate for situations in which accuracy or speed are more important, at least for more simple discriminations, but more exploration is necessary.

Differences in speed–accuracy focus can be problematic for capacity coefficients. Assessment functions (Donkin, Little, & Houpt, 2014; Townsend & Altieri, 2012) are a variation on the capacity coefficient that can ameliorate this problem; however, no inferential statistics are available for the assessment function so we only reported capacity coefficients. We did calculate assessment functions and in all cases, the visual patterns matched our conclusions drawn from the capacity coefficients. These data indicate no significant speed–accuracy impact on processing efficiencies for either algorithmic or cognitive fusion.

## Experiment 2

In Experiment 1, we obtained clear results indicating limited capacity for extracting information from multi-sensor imagery, with both cognitive and algorithmic fusion. The results regarding architecture were less clear and our goal in Experiment 2 was to obtain more robust results from the SIC and MIC analyses. There are a number of potential reasons for the variability across subjects in the SIC results and the relatively weak evidence indicated by the MIC test. First, many participants’ data were not usable due to the lack of survivor function ordering that is necessary for SIC analyses. This meant that there were very few SIC/MIC combinations available from which to draw conclusions. Hence, we doubled the number of participants for Experiment 2. Second, participants in Experiment 1 may not have settled on a particular strategy and, hence, their data may represent a mixture of parallel and serial processing. To address this issue, participants in Experiment 2 had 8 days of experience with the single and fused imagery before we collected data for the SIC/MIC. Furthermore, we limited the stimuli to the facing stimuli from Experiment 1.

For the 8 days of training, we added noise to every LWIR and visible image to slow down the processing of the image information and allow for improvements in performance over the course of training as more efficient strategies may develop. We did so because in Experiment 1 participants demonstrated similar correct RTs in single-sensor conditions (LWIR-only, visible-only) and multi-sensor conditions across both algorithmic and cognitive fusion presentations without any kind of training, strategy instructions, and only brief stimulus presentation times. Therefore, we wanted to slow processing down to leave room for further possible improvements on supplying multiple sensors and after several days of training.

**Fig. 6:**
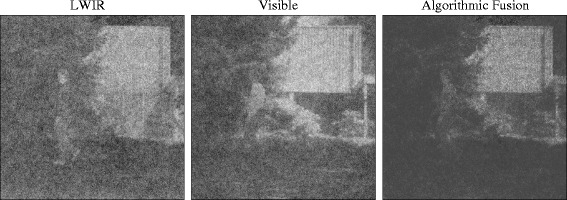
Examples of LWIR, visible, and algorithmically fused images used for the facing discrimination stimuli. In Experiment 2, pink noise was added to every image during the eight training sessions. *LWIR* long-wave infrared

In place of the Gaussian white noise used in Experiment 1, we added pink noise to give a more naturalistic degradation of image quality (Glasgow et al., 2003; Reis, Marasco, Havig, & Heft, 2004). Example stimuli are shown in Fig. 6.

Finally in Experiment 2, we measured only the SIC/MIC for cognitive fusion. Although we did measure capacity coefficients for both cognitive and algorithmic fusion, we did not further examine the algorithmic fusion method because the results from Experiment 1 indicated that selectively influencing each source image would be unlikely if not impossible.

We expected participants to exhibit higher accuracy and lower correct RTs with training. The capacity coefficient represents an improvement in RTs relative to the improvement with single-source images. If training affects not only the perception of each source, but also the efficiency with which participants use the combined information, then we would also expect capacity to increase over training. Alternatively, if there is no additional improvement for the process of combining the information, then the capacity would be stable across training.

Additionally, we hypothesized that participants would use a consistent strategy after training, hence correct RTs would indicate a clear SIC signature (see Fig. 1) and strong evidence from their MIC.

### Method

#### Participants

Twenty individuals (12 male and 8 female) participated in this study. Their ages ranged from 21 to 34 years (*M*=24 years).

#### Materials

Stimuli were selected from Experiment 1 from the facing discrimination stimuli. We chose to use only the actor whom participants from Experiment 1 had indicated was the most clear across the images. To increase the size of the base image set and control for extraneous variation in the images, we edited the images to manipulate the direction the actor was facing and the spatial location of the actor in the image. The editing process involved placing the LWIR and visible image of the actor in ten locations across the image scene. The background scene was averaged across all images to avoid any distortion or aberrations that could influence participant performance. In total, there were 160 stimuli: 2 sensors (LWIR or visible) × 2 directions (left or right) × 2 backgrounds (raw or inverted) × 2 poses (standing or snapshot while walking) × 10 locations (various, ecologically valid, placements across the image). One LWIR-visible pair (same direction, background, pose, and location) was randomly selected for each trial. The LWIR-visible pairs were algorithmically fused to create 80 additional stimuli.

The amount of pink noise was consistent during training within and across participants. We targeted 82% accuracy for each source using the Quest psychometric estimation method with pilot subjects. We chose 82% because it leads to 96% overall accuracy in a UCIP system [ 1−(1−0.86)^2^=0.96].

#### Procedures

Experiment 2 instructions were the same as those used with the facing stimuli in Experiment 1. Participants indicated whether a person was facing to to the left or the right side of the screen (see Fig. 3) using the corresponding mouse button. Participants were told to perform the task as quickly and accurately as possible. At the end of each session, participants were informed of their accuracy in each fusion condition. This feedback was provided to keep participants motivated to improve in performance over the course of the training sessions.

Each participant completed 10 days of 1-hour sessions. The first eight sessions contained trials to compute the capacity coefficient for both cognitive and algorithmic fusion. As with Experiment 1, there were 120 trials per distribution (LWIR-alone, visible-alone, LWIR and visible together) for a total of 720 trials for capacity analysis.

**Fig. 7:**
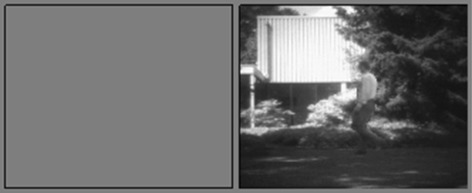
Example of a cognitive fusion presentation of LWIR (absent) and visible (high). The participants were asked to discriminate whether the person was facing to their left or right. The two images were centered and presented within 6.39° of visual angle on a mid-gray background. *LWIR* long-wave infrared

The remaining two sessions required first the estimates of each sensor’s psychophysical thresholds at 82% accuracy by manipulating the amount of pink noise added to the image (120 trials for each sensor for each day) followed by trials required to estimate the SIC (2160 trials in total). The SIC trials consisted of factorial combinations of high (no noise) and low (individualized amount of pink noise) of both the LWIR and visible images. LWIR was always presented on the left and visible on the right. For trials with only one sensor present (e.g., LWIR with high salience, visible is absent), the localization box would appear in place of the image (example shown in Fig. 7).

In cognitive fusion blocks, we fixed the location of where the LWIR and visible images are presented across all trials (LWIR: left of center; visible: right of center) instead of randomly displaying each on the left or right for every trial (as in Experiment 1). This gave operators the opportunity to anticipate where each type of information was going to be presented.

The stimulus presentation duration was extended to 2 seconds across all conditions (algorithmic and cognitive, single- and multi-sensor) to allow the operator to sample all the information from each image and allow strategies of processing the information potentially to improve with time. The LWIR image was always displayed on the left and the visible image was always displayed on the right. Following the stimulus, a blank screen was presented for 500 msec, allowing the participant a total of 2.5 seconds to respond starting from stimulus onset.

### Results

RTs and accuracy with fused imagery were worse than with single-sensor images. Performance on both single- and multi-sensor imagery improved with training; however, the capacity coefficient consistently indicated inefficient performance with both algorithmic and cognitive fusion, lower capacity results for algorithmic fusion than cognitive fusion, and no efficiency improvements with training. Nonetheless, we found strong evidence for parallel and coactive processing strategies with cognitive fusion, both of which are normally associated with efficient processing.

#### Accuracy and mean correct RT analysis

Table 8 gives the results of a 2×8 repeated-measures ANOVA to assess the effects of the number of training sessions completed and the type of fusion (algorithmic or cognitive) for both correct RTs and accuracy for trials with multiple sensors. There was an interaction between training and fusion technique in accuracy, but not RT. For both correct RTs and accuracy, we found a main effect of the number of training sessions completed. There was not a main effect of fusion technique (algorithmic or cognitive) for correct RTs, but there was for accuracy.

**Table 8:** Experiment 2 ANOVA results for the number of training sessions (1–8) and the fusion technique (algorithmic or cognitive) predicting correct response times and accuracy for multi-sensor trials

	Correct response time			Accuracy		
Condition	*F*	*df*	${\eta _{G}^{2}}$	*F*	*df*	${\eta _{G}^{2}}$
Number of training sessions × fusion technique	2.05	7, 133	0.01	2.37*	7, 133	0.02
Number of training sessions	5.03***	7, 133	0.05	19.92***	7, 133	0.32
Fusion technique	2.14	1, 19	0.02	329.18***	1, 19	0.49

${\eta _{G}^{2}}$ generalized eta-squared

* *p*<0.05; ** *p*<0.01; *** *p*<0.001

Although performance clearly improves over training, it is not clear if the efficiency with which individuals use the fused imagery improves from the mean RT and accuracy data. For this information, we need the capacity results, which are presented in the next section.

#### SFT analysis

**Fig. 8:**
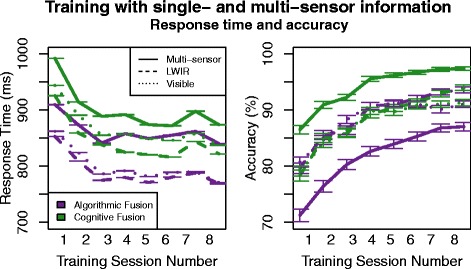
Group-level means of correct RTs and accuracy across days of training. Line type indicates the type of imagery used: fused (*solid*), LWIR (*dashes*), or visible (*dots*). Line color indicates the screen layout of the images: single center-screen images (*purple*) or left/right/both images (*green*). Hence, the algorithmic fusion results (multi-sensor, center-screen) are indicated by *solid purple lines* and the cognitive fusion results (multi-sensor, left/right of center) are indicated by *solid green lines*. Error bars represent the standard error of the mean (Jarmasz & Hollands, 2009). *LWIR* long-wave infrared, *RT* response time

Table 9 gives the results of a 2×8 repeated-measures ANOVA to analyze the effects of training on the efficiency of processing multi-sensor information to predict capacity *z* scores. Participants 12 and 17 were excluded from efficiency comparisons across training sessions because of low accuracy in the early training sessions. Figure 8 illustrates that individual capacity *z* scores with cognitive fusion were less limited than *z* scores with algorithmic fusion [ *t*(143)=−12.19, *p*<.05, *d*=1.45]. Capacity *z* scores become significantly more limited from the first day (Day 1) to the last day (Day 8) of training for both algorithmic fusion [ *t*(17)=3.03, *p*<.05, *d*=1.02] and cognitive fusion [ *t*(17)=2.99, *p*<.05, *d*=0.49].

**Table 9:** Experiment 2 ANOVA results for the number of training sessions (1–8) and the fusion technique (algorithmic or cognitive) predicting group-level mean capacity *z* scores

	*z* score		
Condition	*F*	*df*	${\eta _{G}^{2}}$
Number of training sessions × fusion technique	0.03	1, 16	0.00
Number of training sessions	10.29 **	1, 16	0.05
Fusion technique	21.12***	1, 16	0.53

${\eta _{G}^{2}}$ generalized eta-squared

* *p*<0.05; ** *p*<0.01; *** *p*<0.001

Table 10 indicates the participants whose data passed the selective influence test, the participants’ Houpt–Townsend SIC statistic for both positive and negative deviations from zero, the MIC statistic, and the processing model that predicts their pattern of results. Distributional orderings did not indicate violations of selective influence for 11 participants. Ten of those participants had a significantly positive SIC (*p*<.33). Four of those participants had a significantly positive MIC and significantly negative SIC. One participant had a significantly positive and negative SIC with a non-significant MIC. One participant had a significantly negative SIC. Participant 9 had SIC/MIC results that are not predicted by any of the independent serial/parallel/coactive AND/OR models.

**Table 10:** Cognitive fusion results of Experiment 2 including whether each participant passed the test of selective influence, the Houpt–Townsend statistic (*D* ^+^ and *D* ^−^), the mean interaction contrast (MIC), and the identified processing model

Subject	Pass/fail	*D* ^+^	*D* ^−^	MIC	Architecture
3	Pass	0.349***	0.241**	60.101***	Coactive
9	Pass	**0.160** ^+^	0.182*	**-4.752** ^+^	Ambiguous
10	Pass	**0.190** ^+^	0.071	**48.077** ^+^	Parallel-OR
11	Pass	0.257**	**0.125** ^+^	103.470*	Coactive
13	Pass	0.429***	0.071	152.638***	Parallel-OR
14	Pass	**0.109** ^+^	0.151	16.710	Ambiguous
15	Pass	0.263**	0.225*	51.046*	Coactive
16	Pass	0.230*	**0.154** ^+^	**51.050** ^+^	Coactive
17	Pass	0.198*	0.048	62.970***	Parallel-OR
19	Pass	**0.142** ^+^	0.258**	32.772	Serial-AND
20	Pass	0.041	**0.165** ^+^	−42.617	Ambiguous

*MIC* mean interaction contrast Houpt–Townsend statistic (*D* ^+^, *D* ^−^): ^+^ *p*<0.33; * *p*<0.05; ** *p*<0.01; *** *p*<0.001

MIC: ^+^ *p*<0.33; * *p*<0.05; ** *p*<0.01; *** *p*<0.001

Bold *D* ^+^ and *D* ^−^ statistics indicate a significant Houpt–Townsend statistic at *p*<0.33

With the hierarchical Bayesian MIC analysis, we found good evidence for a positive MIC at the group level ($\hat {p}_{\text {posterior}}^{+}=0.73$). The remaining models were equally unlikely ($\hat {p}_{\text {posterior}}^{-}=0.15$; $\hat {p}_{\text {posterior}}^{0}=0.12$). At the individual level, the posterior probabilities supported the conclusions drawn from the Houpt–Townsend statistic of positive and negative deviations of the SIC (Table 10). The most likely model for nine participants had MIC>0 with MIC=0 as the second most likely. Among those participants, the ratio of posterior odds ranged from 4.5 to 70.0, indicating strong to decisive evidence for each individual. Participant 9 had the most likely positive MIC with MIC<0 second most likely. Participant 20 had a most likely negative MIC with MIC>0 second most likely. Both Participants 9 and 20 had minimal evidence in favor of the most likely model, with a ratio of posterior odds of 1.9 and 2.0 over the next best model, respectively.

### Discussion

In Experiment 2, our aim was to produce consistency within an individual and across people in the processes involved with multi-sensor information. We found nearly identical capacity results with those of Experiment 1 despite the several experimental changes: (1) increased experience with multi-sensor imagery, (2) realistic degradation of image quality with pink noise, (3) longer stimulus presentation time, and (4) fixing LWIR to the left-hand side of the screen and visible to the right-hand side. Even with many experimental changes, we consistently found limited workload capacity with both algorithmic and cognitive fusion. Similarly, the discrepancy between single- and multi-sensor performance with algorithmic fusion was much larger than cognitive fusion. Likewise, we found lower capacity results for algorithmic fusion than cognitive fusion.

When participants had undergone training, there were clear results indicating processing architecture from SIC analyses. We found group-level evidence of parallel-OR or coactive processing (the MIC cannot distinguish between these processing strategies). The ability to process both images in parallel leaves opportunity for facilitation in performance from the redundancy speed-ups across the two images (Kahneman, 1973; Pollatsek et al., 1984).

Over the course of training, performance improved for all single- and multi-sensor conditions. These raw RT results cannot discriminate whether the multi-sensor performance improvement was due to better use of single-sensor images or improvements in the *integration* of the sensor images. By applying the capacity coefficient, it was clear that integration of multi-sensor imagery did not improve with training, and in fact may have degraded.

Despite limited capacity results, we still find evidence for efficient processing strategies. SIC and MIC results from the cognitive fusion conditions indicate clear evidence against serial processes, in favor of parallel-OR or even coactive processing. Although we could not draw conclusions from the algorithm-fused imagery, we assumed serial processing of each source was highly improbable, and the process is more likely a type of coactivation. Thus, the limited capacity results are not due to inefficient serial processing of information. For cognitively fused imagery, the available processing capacity could be divided between the two sources of information and in turn slow down the processing of the individual sensors or the information provided from each sensor inhibits processing of the alternative. For algorithm-fused imagery, limited capacity results may result from inhibition that degrades sensor integration in the overall composite image.

## General discussion

Across the two experiments, we found strong evidence of limited capacity for both algorithmic and cognitive fusion. Although in some cases RTs were faster with fused imagery, they were not as fast as our model predicted given the redundant information across the two sources. Despite the mixed effects we found with raw RTs, the capacity coefficient indicated algorithmic fusion led to more limited capacity performance than cognitive fusion, despite requiring participants to attend to only one image. These capacity results were consistent across a variety of manipulations: stimuli (facing or pointing), difficulty (no noise or pink noise), viewing duration, and variability in single-sensor image placement on the screen (random or predictable).

Image fusion may have the best results when each sensor alone does not supply redundant information; rather, only the configural combination of the information allows for correct decision-making (Klein et al., 2006; Neriani et al., 2008). For instance, Toet et al. (1997) found performance improvements with algorithmically fused LWIR and visible images, contradictory to our findings. The task used in Toet et al. (1997) was tailored specifically to utilize both visible and LWIR information. The participants were asked to determine the position of a person relative to an environmental object (i.e., fence, walkway, or tree). Therefore, to identify correctly the spatial location, the participant must take advantage of unique information from each sensor. Follow-up studies should consider performance comparisons across multi-sensor information presented with algorithmic and cognitive fusion when the individual sensors each supply unique useful information to the observer.

In many cases, it may be difficult to determine a priori the extent to which task-relevant information is redundant across sensors. There is some promise in the recent work by Bittner (2015), which uses response classification (e.g., Ahumada, 2002, Ahumada & Lovell, 1971) to assess the unique information used to make a decision from each sensor image. Response classification uses noise masking to identify the useful information in each single-sensor and multi-sensor image for an observer to make a decision. Clusters of pixels can determine what unique features of each image carry task relative details.

### Algorithmic fusion

Based on the existing research with algorithmic image fusion, we expected fusion would provide, at a minimum, equally efficient processing as an UCIP model. However, our results indicate just the opposite in that it has been an assumption that multi-spectral fusion can enhance both speed and accuracy performance compared to individual sensor images. This discrepancy is partially due to alternative methods of analysis. For some conditions, the traditional analyses of RTs would indicate a benefit in performance with cognitive fusion compared to either single sensor alone (Fig. 5). While it seems as if performance is enhanced with the side-by-side presentation, these RT speed-ups are not faster than what can be attributed to what is expected when completing a task that only demands one source and the fastest of the two can be sampled on each given trial (i.e., statistical facilitation; Raab, 1962).

Some previous research based on traditional analyses has suggested that algorithmic fusion, at best, performed just as well as individual sensor performance and potentially hinders performance or situational awareness (Krebs & Sinai, 2002; Steele & Perconti, 1997; Krebs, Scribner, Miller, Ogawa, & Schuler 1998). In those studies and our current work, it is possible that the quality of information in the algorithmically fused image was degraded compared to the individual sensor images. Even if the fused image were of equal quality to one or other of the original images, it would not be sufficient to achieve unlimited capacity performance because there would be no opportunity for redundancy gain. The algorithmically fused image would need to have *better* information quality than either single-source image.

The potential reduction in image quality may be because no consideration of the task or stimuli was used in choosing the particular algorithm. If task-specific image enhancement techniques are not utilized, task-relevant information may be filtered out in the fusion (Dixon et al., 2006; Toet & Hogervorst, 2012). Ideally, the choice of algorithm should attempt to adjust to particular task demands and environmental constraints to obtain optimal scene information (e.g., Yong, Weiqi, & Rui, 2010); however, when systems are designed for general use, the task many not be known in advance.

### Cognitive fusion

For cognitive fusion, we found RT speed-ups for some conditions when comparing an individual sensor image to the presentation of both images side-by-side. However, those speed-ups were not significantly faster than our predicted model baseline. Limited capacity may result from any violation of the baseline assumptions: unlimited capacity, independence, or parallel processing. By using careful experimental control in Experiment 2, we saw strong evidence for parallel (even coactive) processing, leaving two potential explanations for limited capacity with cognitive fusion. Although the capacity coefficient cannot directly distinguish between violations of independence and workload, we can speculate about the potential underlying mechanisms using previous research in conjunction with our findings: (1) There could be a limitation of workload capacity or (2) there could be dependencies between processing of the two sources of information (Eidels et al., 2011). Although the first is possible, there would have to be an extreme workload capacity limitation to overcome the benefits of coactivation (cf. Townsend & Wenger, 2004). In favor of the latter, McCarley & Krebs (2006) used general recognition theory (Ashby & Townsend, 1986) and found the perceptual dimensions of algorithmically combined imagery are nonseparable. For future research, we are interested in investigating cognitive fusion with general recognition theory as well.

## Conclusions

We demonstrated that SFT aids in assessing various display alternatives by providing additional information about how an operator processes the information in each comparison of interest. We found strong evidence for limited capacity processing of both algorithmic and cognitive fusion of multi-sensor imagery. Despite requiring attention to only a single composite image, algorithmic fusion resulted in more limited capacity than cognitive fusion across several experimental manipulations. Algorithmic fusion may be beneficial only when particular image preprocessing techniques can maximize the strengths of the algorithm given the stimulus environment.

While training participants with the task and imagery can reduce RTs and increase accuracy for both single-source images and algorithmically or cognitively fused images, the efficiency with which participants combine the information does not improve. This lack of efficiency improvement was evident with both algorithmic and cognitive fusion. Despite the consistent inefficiency, individuals can simultaneously process multiple sensor images in parallel.

For unknown task environments, presenting all the information to the operator gives them the opportunity to decide what is useful given the task. However, a multi-sensor display may be beneficial only when each single sensor provides unique useful information to contribute to correct decision-making. System designers should not eliminate the potential for using display methods that provide all the information while minimizing the operator’s invested attentional resources.

## Endnote

^1^In fact, some authors define coactive processing by violations of the race model inequality, an upper bound on parallel processing with context invariance (cf. Miller, 1982).
